# Management of Achilles and patellar tendinopathy: what we know, what we can do

**DOI:** 10.1186/s13047-020-00418-8

**Published:** 2020-09-29

**Authors:** Rocco Aicale, Antonio Oliviero, Nicola Maffulli

**Affiliations:** 1 Department of Musculoskeletal Disorders, Faculty of Medicine and Surgery, University of Salerno, 84084 Baronissi, Italy; 2 Clinica Ortopedica, Ospedale San Giovanni di Dio e Ruggi D’Aragona, 84131 Salerno, Italy; 3 Centre for Sports and Exercise Medicine, Queen Mary University of London, Barts and the London School of Medicine and Dentistry, Mile End Hospital, 275 Bancroft Road, London, E1 4DG England; 4 Institute of Science and Technology in Medicine, Keele University, School of Medicine, Guy Hilton Research Centre, Thornburrow Drive, Hartshill, Stoke-on-Trent, ST4 7QB England

**Keywords:** Tendinopathies, Achilles Tendinopathy, Patellar tendinopathy, Tendon, Graft, Arthroscopy, Tenotomy

## Abstract

Tendinopathies are challenging conditions frequent in athletes and in middle-aged overweight patients with no history of increased physical activity. The term “tendinopathy” refers to a clinical condition characterised by pain, swelling, and functional limitations of tendons and nearby structures, the effect of chronic failure of healing response. Tendinopathies give rise to significant morbidity, and, at present, only limited scientifically proven management modalities exist. Achilles and patellar tendons are among the most vulnerable tendons, and among the most frequent lower extremity overuse injuries. Achilles and patellar tendinopathies can be managed primarily conservatively, obtaining good results and clinical outcomes, but, when this approach fails, surgery should be considered. Several surgical procedures have been described for both conditions, and, if performed well, they lead to a relatively high rate of success with few complications. The purpose of this narrative review is to critically examine the recent available scientific literature to provide evidence-based opinions on these two common and troublesome conditions.

## Background

In the United Kingdom, soft tissues disorders have a prevalence of 18 cases for 1000 individuals, and account for 40% of new rheumatology consultations [1]. Tendons can undergo degenerative and traumatic processes. The most vulnerable tendons are those of the rotator cuff, the long head of the biceps, the wrist extensors and flexors, the adductors, the posterior tibial tendon, the patellar tendon and the Achilles tendon, with tendinopathy commonly secondary to overload [2], though one third of patients with these pathologies do not practice regular physical activity [3].

Tendon injuries can be acute or chronic and caused by intrinsic (age [4], body structure [5], nutrition [6], metabolic diseases [7, 8], genetics [9, 10]) or extrinsic (excessive [2, 11], fatigue and improper [11] loading disuse [12] and external damage [13, 14]) factors, alone or in combination [1]. In acute trauma, extrinsic factors predominate, whilst in chronic cases intrinsic factors also play a role. These factors are associated with the onset of overload pathology of tendons, though there is not a specific cause - effect relationship Table 1.

**Table 1:** Types of tendon collagens

***Types of collagenes***	***Quantity***	***MW***	***Localization***
***Type I***	97–98%	95.000	Endotenion, epitenion, paratenion, MJT
***Type II***	0,2-0,8%	95.000	OTJ cartilage
***Type III***	1–1,5%	95.000	Endotenion, paratenion,, MTJ
***Type IV***	< 0,2%	180.000	MTJ
***Type V***	< 0,2%	300.000	MTJ

*MW* Molecular weight, *MTJ* Musculotendon Junction, *OTJ* Osteotendinous Junction

Microscopic examination of abnormal tendon tissues normally shows a non-inflammatory process [15] with disordered arrangement of collagen fibres, increased vascularisation [16], and poor tendency to healing [17]. An angioblastic reaction is present, with a random orientation of blood vessels, sometimes at right angles to collagen fibres [18]. Inflammatory lesions and the presence of granulation tissue are uncommon and, if present, they are associated with tendon ruptures [17]. Six different subcategories of collagen degeneration have been described, but usually degeneration is of either mucoid or lipoid variety [19]. The characteristic hierarchical structure of collagen fibres is also lost [11]. Furthermore, in the paratenon, mucoid degeneration, fibrosis, and vascular proliferation, with a slight inflammatory infiltrate, have been reported [20]. In 163 patients (75% of whom participated in non-professional sports, particularly running) with classical symptoms and signs of Achilles tendinopathy (AT) for a median of 18 months, changes in collagen fibres’ structure, with loss of the normal parallel bundles, were evident [21]. The areas of altered collagen fibre structure and increased interfibrillar ground substance exhibit an increased signal at magnetic resonance imaging (MRI) [1, 18], and are hypoechoic on ultrasound (US) [22] (Table 1).

The aim of the present narrative review is to critically examine the recent available scientific literature to provide an evidence-based opinion regarding these two clinical syndromes, which are the most common in athletes population with high economic and social relevance, and are not easy to treat.

## Achilles tendinopathy

AT is a common cause of disability in many athletes for the continuous, prolonged and intense functional demands imposed on the Achilles tendon [23], and is common in runners and athletes participating in racquet sports, track and field, volleyball, and soccer [24, 25].

To date, the incidence and prevalence of AT remain non-established, given the lack in scientifically sound epidemiological data [26]. AT is common in athletes, accounting for 6–17% of all running injuries [27, 28], and athletes who participate in repetitive impact physical activities such as running and jumping present an incidence and a lifetime prevalence, respectively, of 9 and 52% in recreational runners [29, 30].

The etiopathogenesis of AT remains unclear but is currently considered multifactorial, and an interaction between intrinsic and extrinsic factors has been postulated [11]. Changes in training pattern, poor technique, previous injuries, footwear, and environmental factors, such as training on hard, slippery, or slanting surfaces, are extrinsic factors that may predispose the athlete to AT [11]. However, also dysfunction of the gastrocnemius soleus, age, body weight and height, pes cavus, marked forefoot varus, and lateral instability of the ankle have been reported as risk factors [11]. Several other factors may play an important role in the etiopathogenesis of tendinopathies such as drugs (i.e fluoroquinolones, in particular, ciprofloxacin, and corticosteroids [14, 31]), imbalance in MMPs activity in response to repeated injury or mechanical strain [32–36], metabolic diseases (i.e. diabetes [37–40]) and/or genetic predisposition [41–43].

AT is clinically characterised by pain and swelling, in and around the tendon, mainly arising from overuse, but often presenting in middle aged overweight individuals with no history of increased physical activity [10]. AT can be categorised as insertional and non-insertional, with different underlying pathophysiology and management options [23, 44, 45].

Pain is the most common AT symptom, but it is not understood how pain arises [6]: it may originate from both mechanical and biochemical causes [46]. Pain typically occurs at the beginning and a short while after the end of a training session. As the pathologic process progresses, pain may occur during the entire exercise session, and it may interfere with activities of daily living [40].

At clinical examination, patients commonly report pain 2 to 6 cm above the insertion of the tendon into the calcaneus [47]. Commonly used and reliable clinical diagnostic tests for Achilles tendinopathy are palpation of the area to ascertain whether pain is elicited, the painful arc sign, and the Royal London Hospital test [48].

Diagnostic imaging, such as plain radiography, US and MRI, may be required to verify a clinical suspicion or to exclude other musculoskeletal disorders [49]. The management of AT lacks evidence-based support, and patients with AT are at risk for long-term morbidity with unpredictable clinical outcome [50]. The management is primarily conservative, and many patients show good outcomes. However, if conservative management fails, surgery is recommended after 6 months of conservative management [51, 52]**.**

### Conservative management

Despite morbidity associated with AT in athletes, management is far from scientifically based, and many of the therapeutic options reported are lacking hard scientific background [53, 54]. In the last few decades, several non-operative treatments modalities have been introduced, with an increasingly relevant role of local drug injections (such as sclerosing agents, corticosteroids, and high-volume image guided injections (HVIGI)) and physical therapy (i.e. shockwave and ultrasound therapy).

Cryotherapy has been regarded as a useful intervention in the acute phase of AT; however, recent evidence in upper limb tendinopathy indicates that the addition of ice did not offer any advantage over an exercise program consisting of eccentric and static stretching exercises [55].

Nonsteroidal anti-inflammatory drugs (NSAIDs) are commonly used, even though AT is not regarded as a classical inflammatory condition. Although NSAIDs may provide some pain relief, they do not actually result in sustained improvement in the healing process [56].

Sclerosing injections can be an option, but contrasting results have been reported [6]. HVIGI injections likely produce local mechanical effects, causing the neovascularity to stretch, break, or occlude, obtaining pain relief given the destruction of sensory nerves [26]. Several substances have been investigated and injected in and around tendons including normal saline, corticosteroids, and local anaesthetics [57, 58], but it is not possible to draw firm, evidence-based conclusions on their effectiveness [6].

Exercise programs with both eccentric and concentric exercises are widely used as first line management of AT, and no studies report adverse effects [6]. Eccentric exercises are superior to wait-and-see treatment [59], and both eccentric and concentric exercises could be considered as equally good for patients with AT. However, given the lack of high-quality studies with clinically relevant results, no strong conclusions can be made regarding the effectiveness of eccentric training (compared with control interventions) in relieving pain, improving function or achieving patient satisfaction [6]. The treatment regime most commonly used comprises 3 sets of 15 repetitions, carried out twice daily, 7 days a week for 12 weeks.

Conservative treatment with shockwave therapy is proving successful, and moderate evidence indicates that ESWT is more effective than eccentric loading for insertional AT [60] and equal to eccentric loading for midportion AT in the short term. Additionally, there is moderate evidence that combining ESWT and eccentric loading in midportion AT may produce superior outcomes to eccentric loading alone [61]. However, the randomised controlled trials on this subject are statistically and clinically heterogeneous, making conclusions from pooled meta-analyses difficult to interpret [6]. Ultrasound therapy is a widely available and frequently used electrophysical agent in sports medicine, but systematic reviews and meta-analyses have repeatedly concluded that there is insufficient evidence to support a beneficial effect of ultrasound at the dosages currently being used in clinical practice [6].

Recent evidence in patients with AT demonstrated strength deficits in the triceps surae of the affected limb compared with the uninjured side or with an asymptomatic control group [62]. When clinicians approach AT, they may need to optimise rehabilitation, implementing a regimen of calf muscle eccentric exercises with heel lifts. These are effective to decrease pain, improve ankle function, and reduce joint dorsiflexion and the strain on the Achilles tendon [63]. Heel lifts reduce tensile loads on the Achilles tendon, counteracting the effect of footwear observed in the above-mentioned studies, and supporting the addition of orthotic heel lifts to footwear in the rehabilitation programme [64].

### Surgical management

In 24 to 45.5% of patients with AT, conservative management is unsuccessful, and surgery may be recommended, generally, often after 6 months of non-operative treatment [65, 66]. However, long-standing AT is associated with poor postoperative results, with a greater rate of reoperation being required before reaching an acceptable outcome [67]. Open surgery for tendinopathy of the main body can be considered, using multiple longitudinal tenotomies, which can be implemented with a side-to-side repair and tendon augmentation or transfer, if significant loss of tendon tissue occurs. In chronic Achilles non-insertional tendinopathy, minimally invasive management can be performed.

**Fig. 1:**
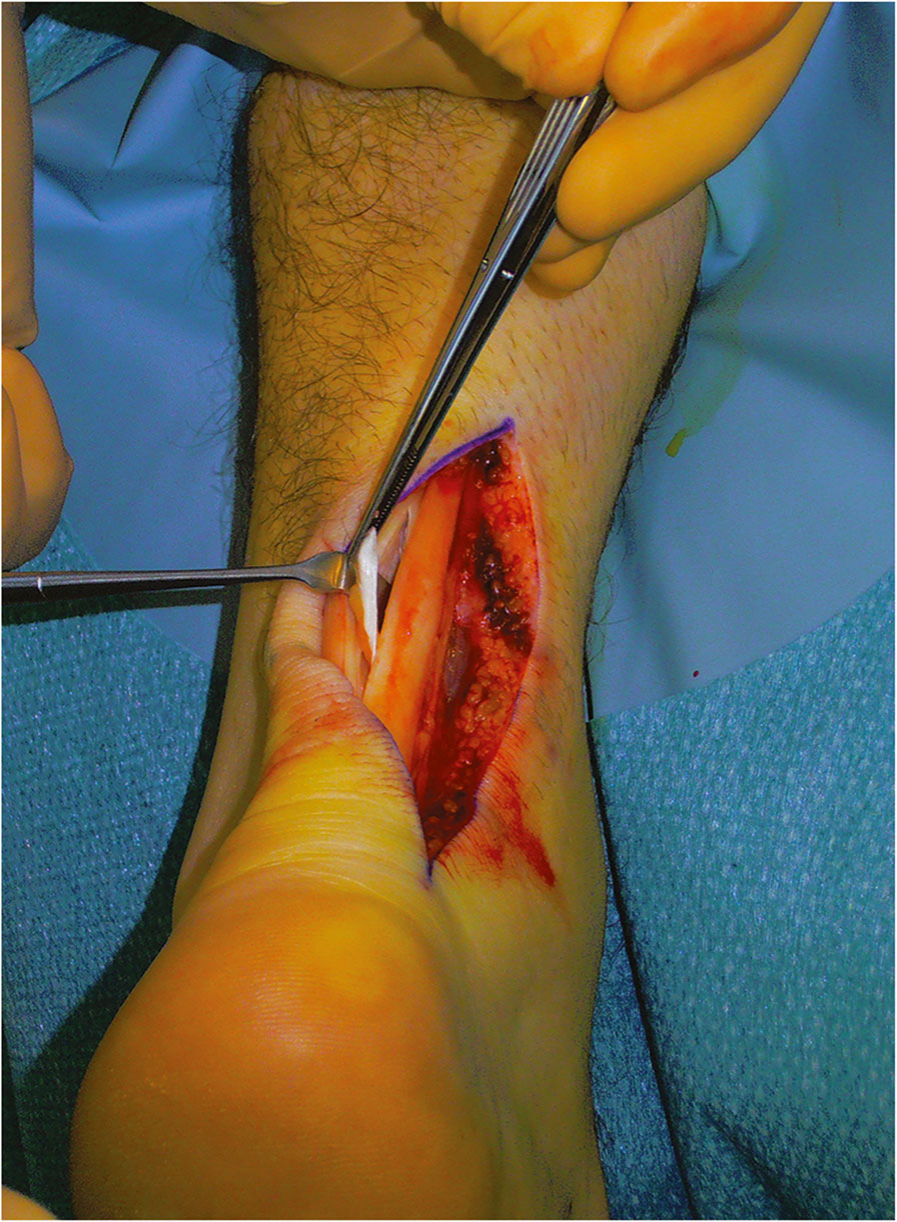
Open surgery for tendinopathy of the main body of the Achilles tendon. The tendinopathic tissue is identified and then excised

Under local, regional or general anaesthesia, the patient is placed prone with the ankles clear of the operating table with a tourniquet, if used, applied to the exsanguinated limb and inflated to 250 mmHg [40]. Generally, the longitudinal incision is made on the medial aspect of the tendon to avoid injury to the sural nerve and short saphenous vein [6]. Based on preoperative imaging studies, the tendon is incised sharply in line with the tendon fibre bundles. Tendinopathic tissue can be identified as it generally has lost its shiny appearance, and frequently contains disorganised fibre bundles that have more of a “crabmeat” appearance: this tissue is sharply excised [6] (Fig. 1). The remaining gap can be repaired using a side-to-side repair, but in our practice, we leave it unsutured. If significant loss of tendon tissue occurs during the debridement, a tendon augmentation or transfer can be considered. The limb is immobilised in a below-knee synthetic weight-bearing cast with the foot plantigrade [40]. Rehabilitation is focused on early motion and avoidance of overloading the tendon in the initial healing phase [6].

**Fig. 2:**
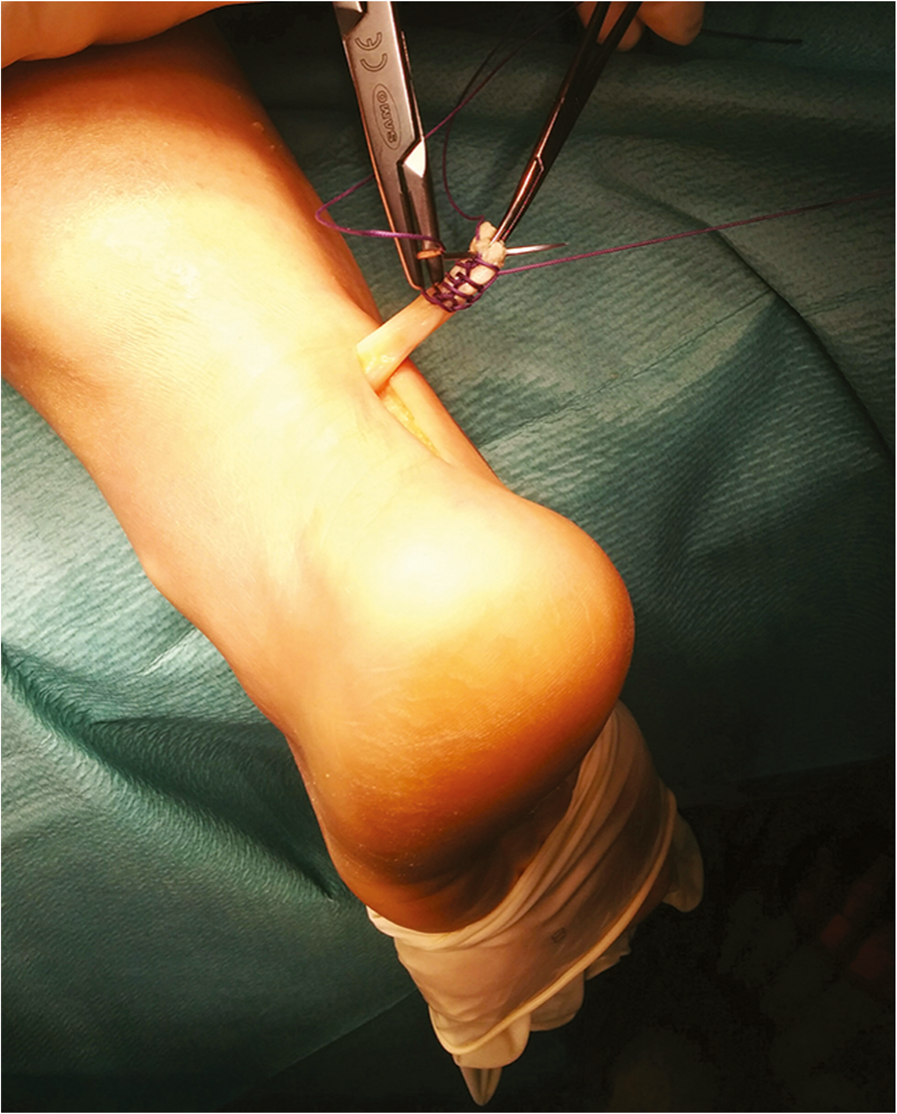
Autograft reconstruction for chronic Achilles tear. The Flexor hallucis longus tendon graft was harvested through a longitudinal medial incision along the distal portion of the Achilles tendon

The surgical procedure described above is relatively straightforward, but on occasion it may require concomitant transfer of tendon tissue to reinforce the weakened tendon [6]. The peroneus brevis, the ipsilateral free semitendinosus, and the flexor hallux longus tendons can be used as tendon grafts [68–70] (Fig. 2).

When conservative management has failed, another less invasive option is multiple percutaneous longitudinal tenotomies which can be used in patients with isolated tendinopathy with no involvement of the paratenon and a well-defined nodular lesion less than 2.5 cm long [71]. If multiple percutaneous tenotomies are performed in the absence of chronic paratendinopathy, the outcome is comparable to that of open procedures [6].

This procedure can be performed in the clinic under local anaesthesia without a tourniquet, but it is important to be careful, since even in minimally invasive procedures complications are possible. The tendon is accurately palpated, and the area of maximum swelling and/or tenderness marked, and checked by US scanning [6]. A #11 surgical scalpel blade is inserted parallel to the long axis of the tendon fibres in the marked area in the centre of the area of tendinopathy. The cutting edge of the blade points caudally and penetrates the whole thickness of the tendon [40]. During this procedure, full passive ankle flexions is made, with the scalpel blade being retracted and inclined several times. Active dorsiflexion and plantar flexion of the foot are encouraged early after surgery [40].

A systematic review of the literature regarding four categories of surgical management (open tenotomy with removal of abnormal tissue, paratenon stripped or not; open tenotomy with longitudinal tenotomy; and percutaneous longitudinal tenotomy) showed successful results in more than 70% of cases for each surgical category [72], but these relatively high success rates are not always observed in clinical practice [72].

**Fig. 3:**
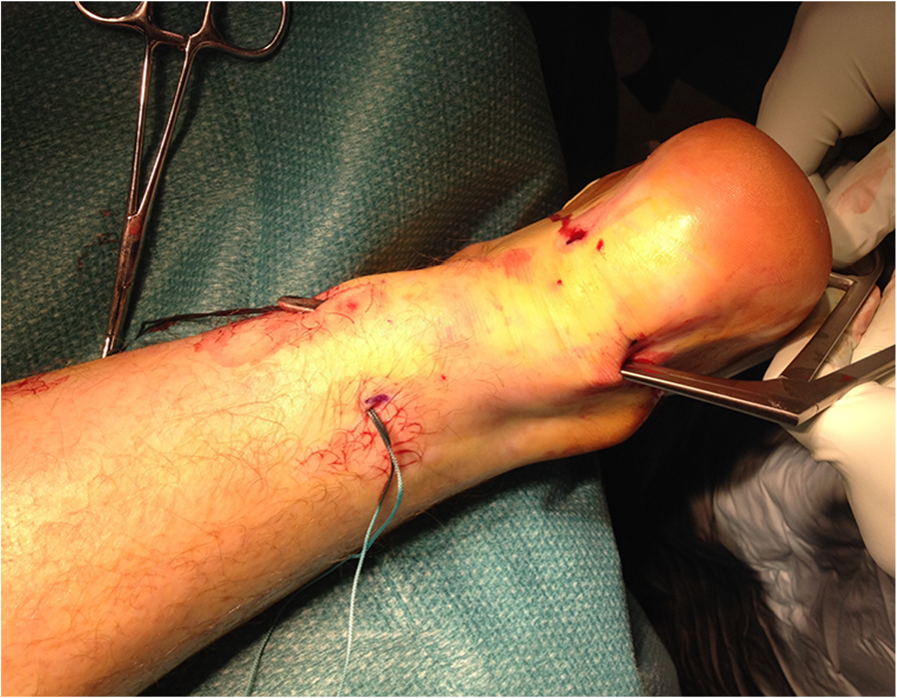
Minimally invasive percutaneous stripping for chronic Achilles tendinopathy. The 4 small incisions are visible, with the surgical instruments passing through

In chronic painful AT, there is neovascularisation outside and inside the ventral part of the tendinopathic area [11, 73]. A minimally invasive management modality can be considered [40] through neovessels stripping from the Kager’s triangle of the Achilles tendon (Fig. 3). This achieves a safe and secure breaking of neovessels and the accompanying nerve supply decreasing pain [6]. The procedure is performed using four longitudinal skin incisions, each 0.5 cm long, and may provide greater potential for the management of recalcitrant AT by breaking neovessels and the accompanying nerve supply to the tendon [6]. The rationale behind this management modality is that the sliding of the Ethibond through the incisions breaks the neovessels and the accompanying nerve supply, decreasing the pain in patients with chronic Achilles tendinopathy [6]. Surgery is successful in up to 85% of patients [65], even though postoperative US examination often shows a widened tendon with hypo-echoic areas. This has led to hypotheses of a possible denervation of the tendon as one of the explanations to the frequently favourable effect of surgery [74].

Non-insertional AT can be also treated with minimally invasive open debridement with resection of the plantaris tendon. This technique has also shown promising results with minimal complications in elite athletes and regular patients with non-insertional AT [75–79]. Whatever the chosen treatment, it is important to stress that patients must be encouraged to weight bear as soon as possible after surgery [6]. A recent systematic review reported that the average success rate of minimally invasive techniques and open procedures is, respectively, 83.6 and 78.9%, while the complication rate was, respectively, 5.3 and 10.5% [80]. The success rates of minimally invasive and open treatments are similar, but there is a tendency for more complications to occur in open procedures. Therefore, minimally invasive surgical treatment would appear to be a useful intermediate step between failed conservative treatment and formal open surgery [81].

## Patellar tendinopathy

Patellar tendinopathy (PT) typically presents with anterior knee pain at to the inferior pole of the patella. The term “Jumper’s knee” was introduced in 1973 by Blazina et al. [82] to describe the condition, as it occurs more commonly in athletes who participate in jumping sports such as basketball and volleyball [83]. Cook et al. [84] found that more than one-third of athletes presenting for treatment for PT were unable to return to sport within 6 months.

Several theories about its pathogenesis, including vascular [85], mechanical [86], impingement–related causes, have been hypothesised, but the most commonly proposed is chronic repetitive tendon overload [87, 88]. The increased strain is located in the deep posterior portion of the tendon, especially with increased knee flexion, between the inferior pole of the patella and the rotation centre of the knee [89]. Microscopic failure occurs at high loads within the tendon and leads alterations at the cellular level, with fibril degeneration which decrease the mechanical properties of the tendon [88]. Studies in vitro and in vivo have shown neovascularisation and increased quantity of proteins and enzymes which can contribute to tendon degeneration [85]. Other studies showed that vascular endothelial growth factor (VEGF) and matrix metalloproteinase (MMP) activity have also been linked to tendon breakdown [36, 90]. A second hypothesised aetiology is the impingement of the inferior patellar pole showed on MRIs during flexion of the knee [91]. The hallmark clinical features of PT consist in pain localised to the inferior pole of the patella [92] and load related pain that increases with the extension of the knee, notably in activities that store and release energy in the patellar tendon [83, 93]. Tendon pain occurs with loading and usually decreases almost immediately when the load is removed [94]. In patients with symptomatic PT, the Royal London Hospital test showed lower sensitivity and higher specificity than manual palpation. Both tests should be performed to formulate a clinical diagnosis of PT. Imaging assessment should be performed as a confirmatory test [48]. PT imaging does not confirm the pain; indeed, intratendinous abnormalities may be observed using US in asymptomatic individuals [95]. Serial imaging is not recommended because, often, symptoms improve without changes in US or MRI [96]. There is no consensus regarding the best management. Avoidance of jumping activities with stretching after physical activity may help in the early phases [92].

### Conservative management

As for Achilles tendinopathy, the first line of conservative management is cryotherapy for its analgesic effect and because it counteracts the neovascularisation process. However, several non-operative treatments modalities have been proposed: oral drugs (NSAIDs and corticosteroids), injections (such as platelet-rich plasma) and physical therapy (i.e. shockwave therapy).

NSAIDs are a mainstay for the management of tendinopathic pain but they are useful only in the short term (7–14 days), in particular in shoulder tendinopathy [97], but there is no long-term benefit. Corticosteroids have been used in various tendinopathies [98–101]. Compared to physical therapy, corticosteroids improve walking pain at 4 weeks, but, while at 6 months physical therapy group had good results, the corticosteroids (CIs) group experienced a relapse [100, 101].

Eccentric exercises (EEs) are the most popular nonoperative treatment but there is no consensus on which the best is [102]. Many EEs protocols are used with different duration and/or frequency, drop squats versus slow eccentric movement, a decline board, and exercising until tendon pain. A study compared primary surgery with an EEs program on a decline board, and at 12 months there was significant improvement in both groups without any significant differences [103]. Visnes et al. [104] compared a decline board program with normal training in elite volleyball players during the playing season and found no significant difference at 6 weeks and 6 months.

An attractive management option is platelet-rich plasma (PRP) injection [105–107], which has some good outcomes [107, 108], but there are no level 1 or 2 studies, and no standards for dosage, injection technique, timing, or number of injections are validated.

Regarding physical therapy, Extracorporeal shock wave therapy (ESWT), generating high strains in the tendon, may produce analgesic benefits through stimulation of tissue healing [109, 110]. There is no consensus on the method of application, generation, energy level, number, frequency of treatments, and the use of anaesthesia [111, 112].

### Surgical management

Approximately 10% of patients are refractory to conservative treatment, and in these patients surgical treatment is indicated [113]. There no consensus on the ideal surgical technique, including whether open techniques are preferable to arthroscopic methods [114–118].

The use of arthroscopy is another possibility, and some surgeons have reported their experience with debridement of the patellar tendon alone [119], while others have described treating both the tendon and bone [120]. Arthroscopic management may be used to debride the adipose tissue of the Hoffa’s body on the posterior aspect of the patellar tendon, to remove the area of neovascularity, to debride the abnormal portion of the patellar tendon, and excise the lower pole of the patella. The surgical approach starts with examination of the knee to exclude coexistent lesions: hypertrophy of the Hoffa fat pad and mucous ligament can often be present, and moderate to severe synovial hypertrophy can be present around the lower pole of the patella [121–123]. The removal of these tissues also allows visualisation of the articular side of the tendon, its insertion to the patella, and the lower pole of the patella. The amount of abnormal patellar tendon is estimated using preoperative MRI and US and used as a guide before surgical debridement. Debridement of the abnormal tendon tissue is carried using an arthroscopic shaver, until abnormal tendon is visualised. Plain radiographs and MRI are used to guide the amount of patella excised, particularly where an inferior spur is present. The inferior pole of the patella is carefully prepared using the radiofrequency probe, and excision of the lower pole of the patella is then performed. Arthroscopic surgery for patients with PT, refractory to nonoperative management, appears to provide significant improvements in symptoms and function [124], with improvements of the International Knee Documentation Committee (IKDC) [125], Lysholm knee score [125], and Victorian Institute of Sport Assessment (VISA) -P scores [126] maintained for 3 years’ follow-up [127]. Recent studies show that partial resection of the distal pole of the patella achieved 90% (18/20) good to excellent results [120], while arthroscopic removal of hypertrophic synovium and fat pad without resection of patellar tendon showed a 76.7% (23/30) return to play rate and 90% good or excellent outcomes [128]. Unfortunately, lack of prospective randomised controlled trials limit the significance of the related studies [124].

**Fig. 4:**
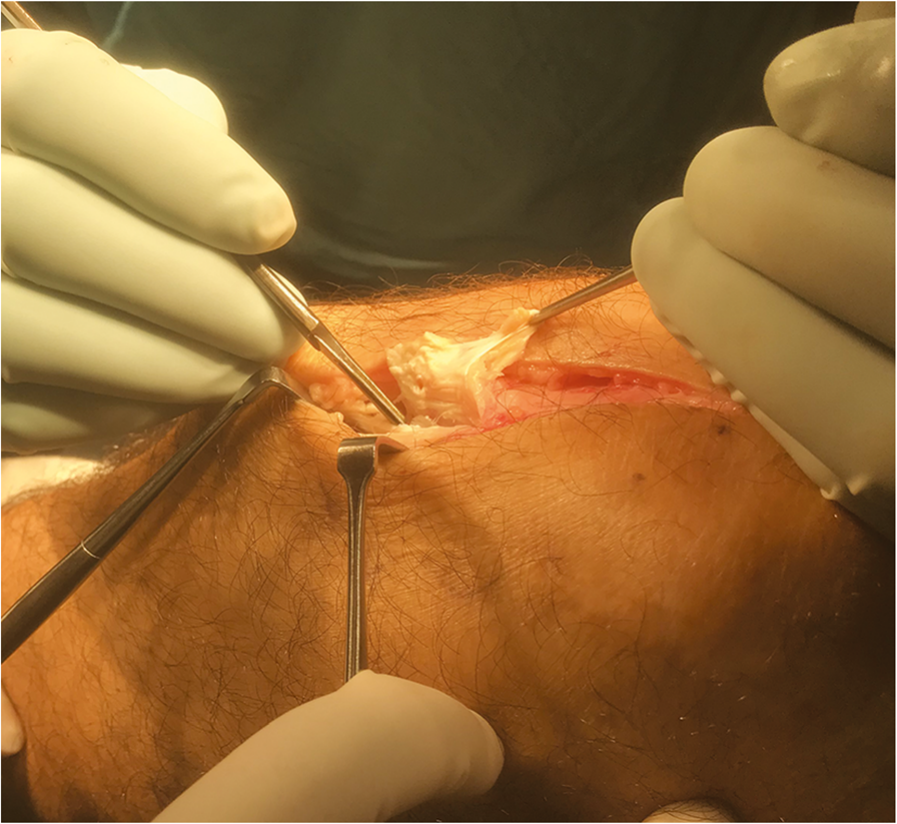
Open surgery for patellar tendinopathy. Excision of the tendinopathic area

Open surgical techniques include opening of the peritendon, removal or drilling of the patellar pole, multiple longitudinal tenotomies and excision of the tendinopathic area [129, 130] (Fig. 4). These are not technically demanding, are reasonably fast to perform and inexpensive, and provide a high rate of good and excellent outcomes in the long term in patients unresponsive to non-operative treatment [131]. Using a midline longitudinal incision and after excision of the paratenon, the tendon is exposed and separated from the Hoffa’s body by blunt dissection. The tendon is palpated to locate any tendinopathic lesions, which usually present as an area of intratendinous thickening. Three longitudinal tenotomies from the lower patellar pole to the tibial tubercle are made, and the tendinopathic areas are excised. The tendon and paratenon are not repaired. A wool and crepe bandage is applied and kept in place for 2 weeks. The tissues excised can be fixed in 10% buffered formalin and sent for histology analysis [132, 133]. Maffulli et al. [131] evaluated the return to sport activity using open technique in two group of patients, one with unilateral and the other with bilateral tendinopathy. At the final follow-up, in both group, the VISA-P scores [126] were significantly improved compared to preoperative values, with no intergroup differences, concluding that this procedure provide a high rate of good and excellent outcomes in the long term.

A recent systematic review reported an average success rate of 87% for the open treatment and of 91% for the arthroscopic surgery, with an average rate of return to sport of 78% after open surgery and 82% after arthroscopic surgery. The average time for return to sport was faster in patients treated arthroscopically compared with open surgery (3.9 vs. 8.3 mouths, respectively) [134]. Moreover, if good to excellent results have been reported after surgical treatment, in about 10% of patients surgery is unsuccessful [135].

**Fig. 5:**
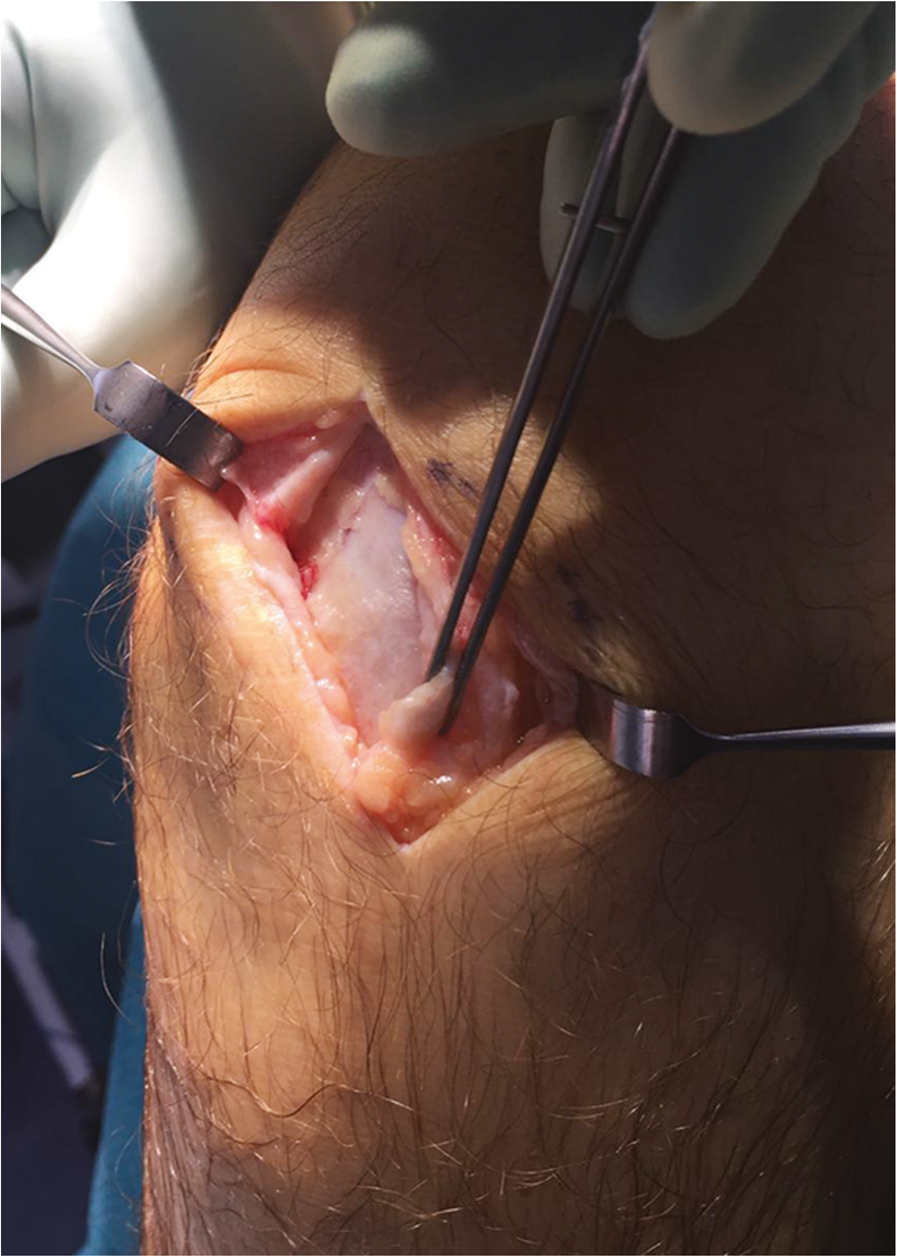
Open surgery for patellar tendinopathy. The patellar tendon is explored and the tendinopathic areas are excised

Refractory PT after surgical treatment involves a small number of patients. Nevertheless, it is serious and debilitating, particularly in young athletes. We consider that a patient is a failure of surgical treatment if they failed to return to sport and are still experiencing pain after at least 1 year of the procedure [135]. Regardless of the first procedure, open or arthroscopic, we use a formal open approach. If the procedure had been performed in an open fashion, surgery is performed through the old incision, with the knee flexed to 90 degrees. The paratenon is opened longitudinally and the patellar tendon is exposed, then, after identification of tendinopathic areas, three longitudinal tenotomies are made (Fig. 5). A wool and crepe bandage are applied and kept in place for 2 weeks. Immediate postoperative mobilisation is recommended with crutches, weight-bearing is allowed as tolerated, and isometric exercises of the quadriceps muscles are encouraged as soon as patients could tolerate them. Patients are reviewed at 2 weeks from surgery, when active mobilisation is encouraged. At 6 weeks, if full active and passive motion have been regained, patients are prompted to start concentric exercises [135].

## Conclusions

The management of tendinopathy remains a major challenge. Advances in operative management are being made and are underpinned by a greater understanding of the pathologic changes of overuse tendon injuries within sport. The lesion is a failed healing response of the tendon, with differences dependent on the site of the lesion. Initially, a nonoperative regimen consisting of physical therapy with eccentric exercises is the mainstay of patellar tendinopathy treatment. Evidence-based guidelines regarding their use are inconclusive. Good outcomes have been obtained in refractory cases in both Achilles tendinopathy and patellar tendinopathy following surgery. However, we need further controlled studies to evaluate and improve novel treatment approaches.

## Data Availability

Not applicable.

## Abbreviations

AT: Achilles tendinopathy
MRI: Magnetic resonance imaging
US: Ultrasound
NSAIDs: Nonsteroidal anti-inflammatory drugs
ESWT: Extracorporeal shock wave therapy
PT: Patellar tendinopathy
VEGF: Vascular endothelial growth factor
MMP: Matrix metalloproteinase
EEs: Eccentric exercises
CIs: Corticosteroids
PRE: Platelet-rich plasma
IKDC: International Knee Documentation Committee
VISA: Victorian Institute of Sport Assessment
HVIG: High-volume image-guided
